# RCCS Bioreactor-Based Modelled Microgravity Induces Significant Changes on *In Vitro* 3D Neuroglial Cell Cultures

**DOI:** 10.1155/2015/754283

**Published:** 2015-01-13

**Authors:** Caterina Morabito, Nathalie Steimberg, Giovanna Mazzoleni, Simone Guarnieri, Giorgio Fanò-Illic, Maria A. Mariggiò

**Affiliations:** ^1^Department of Neuroscience, Imaging and Clinical Sciences, Unit of Functional Biotechnology, Aging Research Center (Ce.S.I.), “G. d'Annunzio” University of Chieti-Pescara, Via dei Vestini 29, 66100 Chieti, Italy; ^2^Interuniversity Institute of Myology, Italy; ^3^Laboratory of Tissue Engineering, Department of Clinical and Experimental Sciences, School of Medicine, University of Brescia, Viale Europa 11, 25123 Brescia, Italy; ^4^Section of Physiology and Physiopathology, Department of Neuroscience, Imaging and Clinical Sciences, “G. d'Annunzio” University of Chieti-Pescara, Via dei Vestini 31, 66013 Chieti, Italy

## Abstract

We propose a human-derived neuro-/glial cell three-dimensional *in vitro* model to investigate the effects of microgravity on cell-cell interactions. A rotary cell-culture system (RCCS) bioreactor was used to generate a modelled microgravity environment, and morphofunctional features of glial-like GL15 and neuronal-like SH-SY5Y cells in three-dimensional individual cultures (monotypic aggregates) and cocultures (heterotypic aggregates) were analysed. Cell survival was maintained within all cell aggregates over 2 weeks of culture. Moreover, compared to cells as traditional static monolayers, cell aggregates cultured under modelled microgravity showed increased expression of specific differentiation markers (e.g., GL15 cells: GFAP, S100B; SH-SY5Y cells: GAP43) and modulation of functional cell-cell interactions (e.g., N-CAM and Cx43 expression and localisation). In conclusion, this culture model opens a wide range of specific investigations at the molecular, biochemical, and morphological levels, and it represents an important tool for *in vitro* studies into dynamic interactions and responses of nervous system cell components to microgravity environmental conditions.

## 1. Introduction

Microgravity modulates numerous features and functions of biological organisms through its effects on physical phenomena, such as hydrostatic pressure in fluid-filled compartments, sedimentation of organelles, and convection processes of flow and heat. These physical parameters can, in turn, directly and indirectly influence cellular and tissue morphology, metabolism and signalling, and, consequently, a wide range of cell functions [1]. Several years ago, it was proposed that gravity is involved in embryonic development, through effects on morphogenesis and organogenesis of the central nervous system and on sensory organs in invertebrates and vertebrates. In particular, when amphibian eggs were fertilised* in vivo* or* in vitro* under microgravity conditions, some abnormalities during embryonic development were observed, even if compensatory mechanisms produced nearly normal larvae [2]. Also, during space flight, signs of neurophysiological impairment have been observed for astronauts, although few studies have been carried out to investigate such effects on the nervous system, in particular at the cellular level [3].

Recently Pani and colleagues reported that neuronal monolayers showed alterations in morphology and viability when exposed to short- and middle-term simulated microgravity in the random positioning machine, while long-term exposures revealed high adaptation of single neurons to the new gravity conditions [4]. Also other neuronal cell models showed morphological and/or cytoskeletal alterations when exposed to simulated weightlessness or during changing gravity [5, 6]. These effects appeared conditioned by the presence of microgravity conditions, and after short-term exposures, under ground-conditions, the cells were able to fully recover their features and the ability to form adherent monolayer cultures [4, 7].

Traditional monolayer cell cultures that are kept under static conditions (two-dimensional (2D) cell culture) have provided great advances in our understanding of the physiological regulatory processes of single cells. On the other hand, the intrinsic complexity of cell-cell extracellular signalling and the remarkable plasticity in the composition and structure of the extracellular matrix have made it very difficult to study these interactions using conventional cell-culture techniques. For these reasons, advanced methods are needed to grow cells while maintaining their native three-dimensional (3D) cytoarchitecture and the specific tissue-like microenvironment. Interestingly, 3D cultures have been shown to favour the maintenance of tissue-specific phenotypes and tissue-like cytoarchitecture. However, an important limitation for long-term culture in three dimensions is the low diffusion of oxygen and nutrients and the absence of a blood supply to the deeper parts of the tissue construct. This is particularly the case for neural cells, and it can result in the appearance of a central core of dead cells [8, 9].

In the 1990s, after the beginning of the many international space programmes, attempts were made to grow 3D cell cultures or tissue explants in particular microenvironments, to test the effects of reduced gravity. Major efforts have been addressed to the building of a system that can reproduce a tissue-like microenvironment* in vitro* and to study the cytoskeletal and nuclear matrix protein interactions during cell exposure to simulated microgravity, as is present in space [10]. Engineers at the US National Aeronautics and Space Administration (NASA) devised a rotating bioreactor, which is a useful device for culturing cells on Earth, as well as in space. Briefly, this monoaxial clinostat (the rotary cell-culture system (RCCS) bioreactor) is a horizontally rotating and fluid-filled culture vessel that is equipped with a gas-exchange membrane that optimises the oxygen supply to the biological samples. Without air bubbles or air-liquid interface, the fluid dynamic conditions inside the culture chamber generate a laminar flow state that greatly reduces shear stress and turbulence, which are hazardous for cell survival. These dynamic conditions provided by the RCCS bioreactor favour spatial colocalisation and three-dimensional assembly of single cells into aggregates [11]. The rotational speed of the culture chamber can be modified to set conditions in which the 3D cell constructs/aggregates also rotate around their own axes, further providing an efficient high mass transfer of nutrient and wastes. When cultured cell aggregates grow in size, the rotational speed of the culture vessel can be increased, to compensate for the increased sedimentation rates. The operational conditions of the RCCS bioreactor can also be adjusted so that the gravitational vectors are randomised up, to reach a modelled microgravity state [12, 13]. In this way, 3D biological samples can remain in a constant orientation, with respect to the chamber wall, and move in near-solid body rotation with the fluid, thus fulfilling the requirements needed to successfully model microgravity conditions [14].

In the present study, we aimed to develop a 3D dynamic* in vitro* neuroglial coculture system, to evaluate the capacity of the cells to reproduce, at least in part, neuronal features. To this end, we used two well-characterised cell lines, GL15 and SH-SY5Y cells, which are astrocyte-like and neuronal-like cells, respectively. The human glioblastoma GL15 cell line is an established* in vitro* astrocyte model that has been functionally characterised by our group and others [15, 16], and these express a typical astroglial phenotype and functions. The human neuroblastoma-derived SH-SY5Y cells are a widely used and well-characterised neuronal cell model that has been extensively used for* in vitro *neurotoxicity testing and has been shown to differentiate towards either adrenergic or cholinergic phenotypes [17–20]. In addition, the human origin of these cell lines makes them an appealing model for basic* in vitro *research studies. Thus, to develop astrocyte-like or neuronal-like* in vitro* models, 3D monotypic cultures (GL15 cells only or SH-SY5Y cells only) were established in a RCCS bioreactor. Of note, it has been demonstrated that cell-cell interactions, as for example, those between glial cells and neurons, are crucial for both glial and neuronal differentiation and developmental processes, as well as for response to neural injury [21, 22]. For these reasons, we also established 3D neuronal/glial heterotypic cultures (cocultures), to more closely reproduce the* in vivo* microenvironment of the nervous tissue and to bridge the gap between* in vitro* systems and animal models. These analyses were also performed under modelled microgravity when the 3D cell aggregates were sufficiently grown in size to adjust to the operational conditions of the RCCS bioreactor, so as to reach a state of vector-averaged microgravity. Under such conditions, their cell morphology, viability, and functional features were analysed and compared.

## 2. Materials and Methods

All of the reagents for cell culture were from Life Technologies (Milan, Italy). The plasticware was from BD Falcon (Sacco, Milan, Italy).

### 2.1. Cell Culture

The SH-SY5Y cell line (from the European Collection of Cell Cultures, supplied through Sigma-Aldrich, UK) and the GL15 cell line were both cultured in Dulbecco's modified Eagle's medium (DMEM) with 10% foetal bovine serum, 100 IU/mL penicillin, 100 *μ*g/mL streptomycin, and 1 mM glutamine. The cells were amplified in monolayers and detached for subculturing using 0.05% trypsin and 0.02% EDTA. SH-SY5Y and GL15 cell cultures, used for experimental assays, were prepared by seeding cells in T75 Falcon flasks, to form 2D static monolayer cultures, or in the RCCS bioreactor to establish 3D cultures subjected to microgravity. Both culture models were cultured in DMEM with 10% foetal bovine serum, 100 IU/mL penicillin, 100 *μ*g/mL streptomycin, and 1 mM glutamine and maintained in the same incubator (5% CO_2_, at 95% humidity) for the same times, and the medium was refreshed twice a week.

### 2.2. 3D Culture in the RCCS Bioreactor

The RCCS bioreactor (Synthecon, Houston, USA) can generate a special microenvironment where high mass transfer is achieved with low shear stress. It is equipped with a cylindrical growth chamber that contains an inner corotating cylinder with a gas-exchange membrane (a 55 mL autoclavable slow-turning lateral vessel) where specific hydrodynamic and physical conditions are attained. The culture of cell spheroids was performed in this device in a 5% CO_2_ incubator at 95% humidity. The horizontally rotating culture vessel was filled with the complete medium (without air-liquid interface to reduce the shear stress). After a defined rotational speed was reached, the cells were cultured under Earth gravity, in a near laminar fluid flow environment (i.e., a free-fall state). Under such conditions, the cells grew in the form of 3D multicellular aggregates [23, 24].

The cell-density seeding for both GL15 and SH-SY5Y cells was approximately 1.5 × 10^6^ cells/mL. The medium was refreshed twice a week. For cocultures, the SH-SY5Y and the GL15 cells were each seeded at a density of 0.75 × 10^6^ cells/mL. The rotational speed of the culture chamber was initially set at between 6 rpm and 8 rpm, and then it was gradually increased as the multicellular aggregates increased in size, to maintain the aggregates in constant equilibrium (i.e., under free-fall conditions).

At the indicated times, the cells were harvested, and according to the experimental conditions required, the multicellular aggregates were either included in Tissue-Tek OCT compound (VWR International Srl, USA) (for* in situ *analysis) or centrifuged at 2300 rpm for 5 min at 4°C, and the resulting cell pellets were kept at −80°C until Western blotting was carried out.

For the embedded aggregates, slices (6 *μ*m to 10 *μ*m) were prepared with a CM1900 cryostat (Leica, Milan, Italy) and processed for cell viability assays or frozen at −20°C for further investigations.

### 2.3. Morphological Analysis

The frozen sections were left to warm up to room temperature and were subsequently incubated for 12 min in Harris' haematoxylin solution, washed twice in water, and incubated for 15 s in Eosin solution. After washing, the sections were dehydrated and mounted with Eukitt mounting medium (Electron Microscopy Sciences). The sections were examined under a Vanox optical microscope (Olympus, Opera Zerbo, Italy).

### 2.4. Cell Viability Assay

The sections were incubated for 15 min at room temperature in a solution containing recombinant Annexin V conjugated to the Alexa 488 fluorophore and propidium iodide (Vybrant kit #2, Life Technologies, Italy) as described by the manufacturer. Moreover, to quantify the total number of cells in the aggregates, 4′-6-diamidino-2-phenylindole (DAPI) was added to this solution at a final concentration of 0.10 *μ*g/mL. The sections were mounted in Prolong antifade medium (Life Technologies) and examined under an inverted fluorescence microscope (Axiovert; Zeiss, Arese, Italy) equipped with an image analyser. Photomicrographs were analysed with the ProImage+ and Scion Image software (http://proimage.software.informer.com/ and http://scion-image.software.informer.com/), to determine the cell viability.

### 2.5. Immunostaining Assay

The frozen GL15 and SH-SY5Y cells in OCT sections were fixed with 3.7% paraformaldehyde at room temperature for 30 min. Slices were then permeabilised with 0.1% Triton X-100 at room temperature for 15 min and incubated for 1 h in 10% bovine serum albumin at room temperature and then for 1 h at 37°C with the primary antibody, followed by 1 h at 37°C with either an Alexa 488- or an Alexa 633-conjugated secondary antibody (Molecular Probes, Milan, Italy). For double staining, the second primary antibody was incubated with the constructs after removal of the first, Alexa 488-conjugated secondary antibody. After the antibody incubation, the cells were washed three times with 0.1% Tween 20, each for 5 min at room temperature. Finally, the nuclei were stained with 1 *μ*g/mL propidium iodide for 30 min. After three washes with phosphate-buffered saline, they were mounted on coverslips and examined.

Primary monoclonal mouse antibodies, neuronal cell adhesion molecule (N-CAM), tyrosine hydroxylase, growth associated protein 43 (GAP43), glial fibrillary acidic protein (GFAP), and S100B were from Sigma-Aldrich (Milan, Italy), and connexin 43 (Cx43) was from Chemicon International Inc. (Temecula, CA, USA).

The fluorescence images were obtained using a Zeiss LSM510 META confocal system (Jena, Germany) connected to an inverted Zeiss Axiovert 200 microscope equipped with a Plan Neofluar oil-immersion objective (40x/1.3 NA).

### 2.6. Western Blotting

Frozen pellets of the GL15 and SH-SY5Y cell aggregates were lysed in cell lysis buffer (50 mM Tris-HCl, 100 mM NaCl, 50 mM NaF, 40 mM *β*-glycerophosphate, 5 mM EDTA, 1% Triton X-100, 200 *μ*M sodium orthovanadate, 100 *μ*g/mL phenylmethylsulfonyl fluoride, 10 *μ*g/mL leupeptin, 5 *μ*g/mL pepstatin A, 10 *μ*g/mL benzamidine, and pH 7.4). After vortexing for 5 min, the samples were centrifuged at 1000 rpm for 10 min at 4°C in a microcentrifuge. The protein content of each supernatant was quantified colorimetrically (Bio-Rad Laboratories Srl, Milan, Italy), and aliquots containing 40 *μ*g protein were added to Laemmli buffer (8% SDS, 10% glycerol, 5% *β*-mercaptoethanol, 25 mM Tris-HCl, 0.003% bromophenol blue, and pH 6.5) and applied to and separated by SDS-PAGE on 7% to 10% SDS polyacrylamide slab gels. Proteins were electroblotted onto hydrophobic polyvinylidene difluoride membranes (Immobilon, Millipore, Milan, Italy) using a tank transfer system (Bio-Rad Laboratories Srl). Transfer efficiency was verified by Ponceau red staining of the blots and Coomassie blue staining of the gels. The SH-SY5Y cell blots were incubated with the following mouse monoclonal antibodies: anti-N-CAM (1 : 100 dilution; Sigma-Aldrich), anti-tyrosine hydroxylase (1 : 1000 dilution; Sigma-Aldrich), anti-GAP43 (1 : 1000 dilution; Sigma-Aldrich), and/or anti-Cx43 (1 : 1000 dilution; Chemicon). The GL15 cell blots were incubated with the following mouse monoclonal antibodies: anti-glial fibrillary acidic protein (GFAP; 1 : 500 dilution; Sigma-Aldrich), anti-S100B (1 : 500 dilution; Sigma-Aldrich), and/or also Cx43 (1 : 1000 dilution; Chemicon). These were then detected by chemiluminescence (ECL plus; GE Healthcare). Moreover, after a membrane-stripping procedure, the GL15 and SH-SY5Y cell membranes were immunostained with a mouse monoclonal anti-actin antibody (1 : 1000 dilution; Sigma-Aldrich).

## 3. Results

### 3.1. Cell Aggregates in the RCCS Bioreactor: Morphology and Viability

#### 3.1.1. GL15 Cells

Initial experiments were performed to establish the most suitable protocol to prepare the cell aggregates. GL15 cells were incubated in the RCCS bioreactor as preinduced cell clusters or as homogeneous cell suspensions that were left to spontaneously aggregate. The preinduced aggregates were obtained using the hanging drop method (see [24]). Both types of aggregates were maintained under conditions of microgravity in the RCCS bioreactor for up to 2 weeks. The single cells spontaneously aggregated within 48 h of culture, although some features of the spontaneous cell aggregates were different compared to the preinduced aggregates.

**Figure 1:**
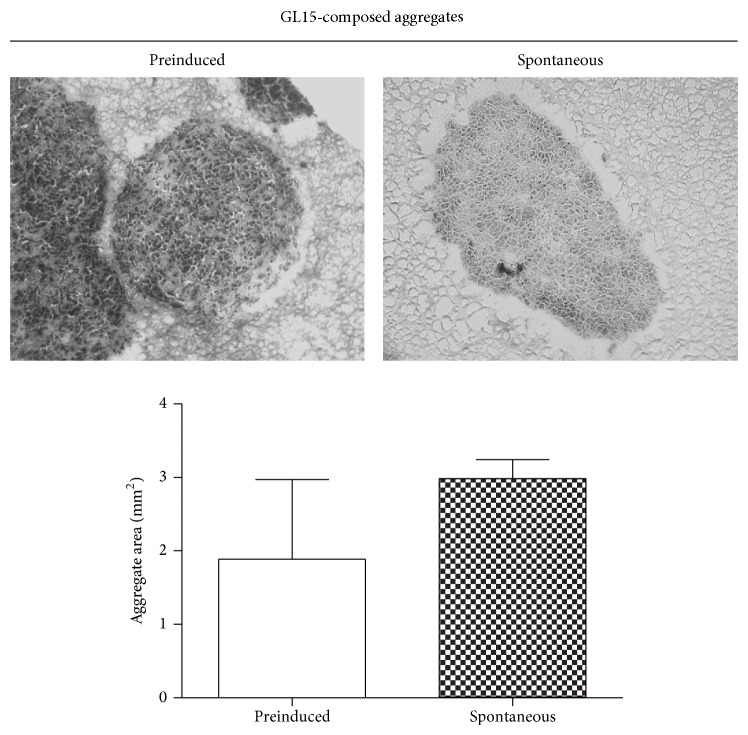
GL15 cell aggregate morphology. Representative images and quantification of sections from preinduced and spontaneously formed GL15 aggregates (as indicated). Data are means ± SEM. *n* = 15 for the averaged areas of the aggregate sections, calculated using the ImageJ software (http://imagej.nih.gov/ij/).

The preinduced aggregates provided relatively uniform clusters, while the spontaneously formed aggregates appeared more irregular in shape. In addition, after 2 weeks in the RCCS bioreactor, the spontaneously formed aggregates showed a trend (not significant) towards a greater mean area (2.98 ± 0.26 mm^2^), compared to that of the preinduced aggregates (1.89 ± 1.28 mm^2^) (Figure 1).

**Figure 2:**
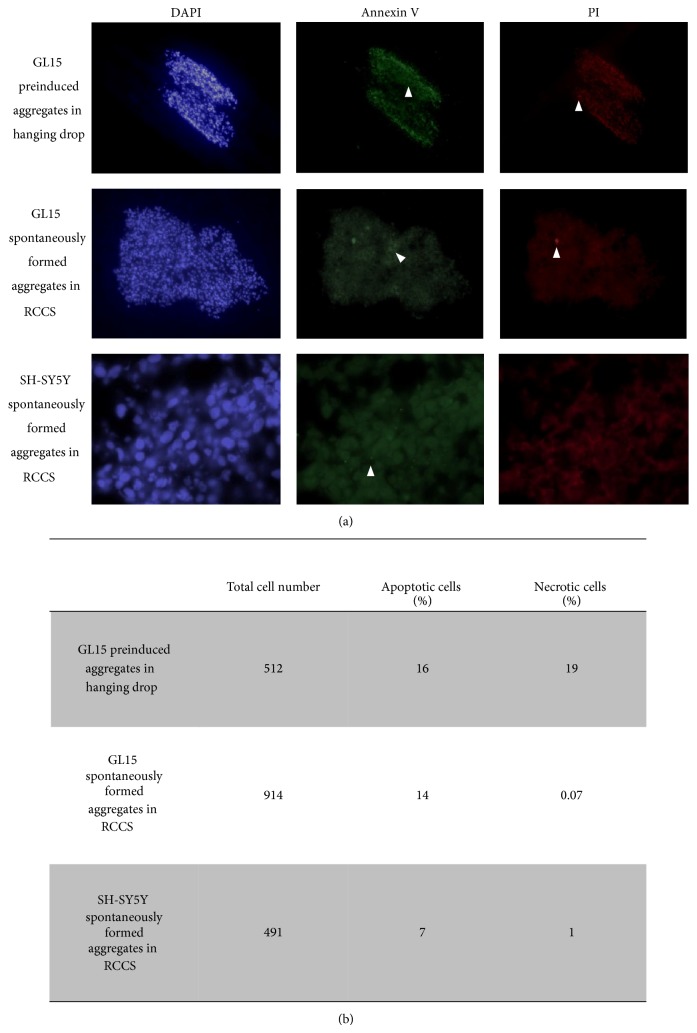
Cell viability assay. (a) Representative images of preinduced and spontaneously formed GL15 aggregates and spontaneously formed SH-SY5Y aggregates (as indicated). Aggregates were stained with DAPI (blue), Annexin V-Alexa 488 (green), and propidium iodide (PI; red). DAPI-positive/Annexin V-Alexa 488-negative/PI-negative cells are healthy; DAPI-positive/Annexin V-Alexa 488-positive/PI-negative and PI-positive cells are considered apoptotic (Annexin V, arrowheads); DAPI-positive/Annexin V-Alexa 488-negative/PI-positive cells are necrotic (PI, arrowheads). (b) Quantification of apoptotic and necrotic cells in aggregate sections. Data derived from 3 independent experiments.

The cell viability in the preinduced aggregates and the spontaneously formed aggregates was also assessed after 2 weeks in the RCCS bioreactor, to determine the apoptotic or the necrotic cells (Figure 2). To this aim, the cells were tested to measure early apoptosis by detecting phosphatidylserine expression revealed by Annexin V binding or necrosis by membrane permeability to the propidium iodide (PI) vital dye. Cells positive to Annexin V green fluorescence signal are known to be apoptotic cells, while those positive to PI red fluorescence signal are necrotic cells; the absence of green or red signal and the nuclear staining with DAPI revealed viable cells. The image analyses of stained cells revealed that some apoptotic cells (Figure 2(a), green fluorescence) were evident at a similar extent in preinduced and spontaneous GL15 aggregates (Figure 2(b)). A relevant amount of necrotic cells (Figure 2(a), red fluorescence) was present in preinduced aggregates compared to spontaneous ones in which necrotic cells were nearly absent (Figure 2(b)). These data revealed the presence of possible stress conditions in the preinduced aggregates, while the spontaneously formed aggregates showed cells that were in a more healthy state. This cell stress might be the result of hypoxic processes in the central core of the preinduced aggregates in particular, potentially due to the static conditions encountered in the hanging drops. For this reason, the rest of the investigations used only the spontaneously formed cell aggregates, and those formed by the GL15 cells are henceforth referred to as the G-aggregates.

#### 3.1.2. SH-SY5Y Cells

Following the same procedure described above for the formation of the G-aggregates, the SH-SY5Y cells were cultured to form spontaneous aggregates in the RCCS bioreactor for up to 2 weeks, and their cell morphology and viability were then assessed. These aggregates formed by the SH-SY5Y cells are henceforth referred to as the S-aggregates.

At the end of the incubation period, the S-aggregates showed variable and irregular shapes, with a mean area of 2.68 ± 0.13 mm^2^. In addition, there were very low levels of apoptotic and necrotic cells (Figure 2(b)), which indicated that these 3D dynamic culture conditions are a suitable method to sustain cell viability also for neuronal-like cells.

### 3.2. Qualitative Analysis of Phenotype-Specific Markers

#### 3.2.1. GL15 Cells

**Figure 3:**
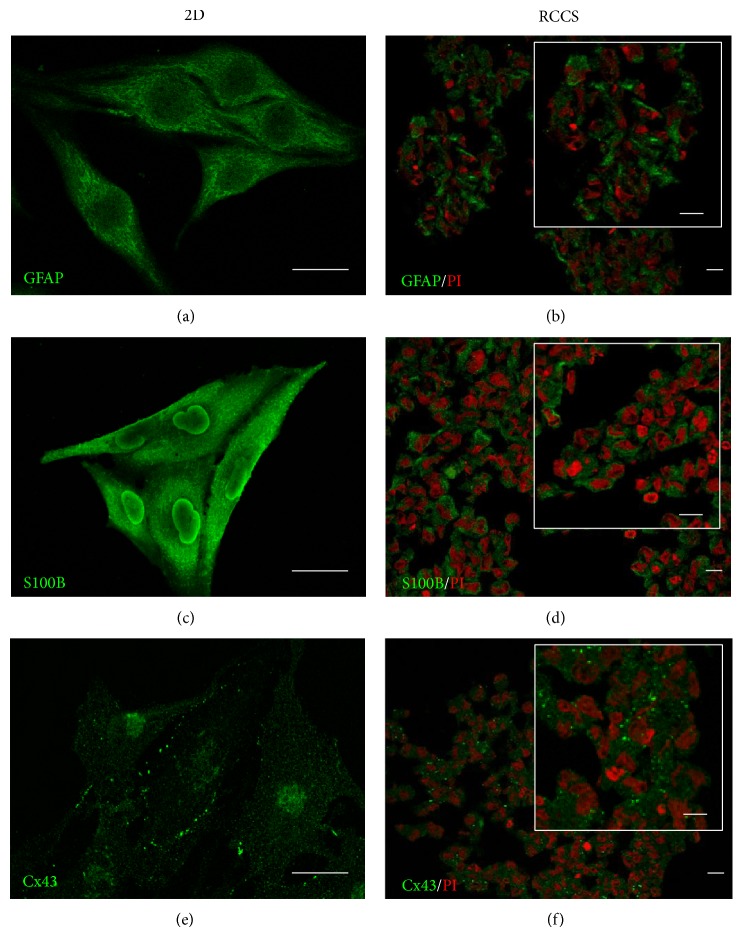
Glial marker localisation in GL15 cells. Representative confocal images of GL15 cells cultured as a monolayer (2D, (a), (c), and (e)) and under the modelled microgravity (RCCS bioreactor, (b), (d), and (f)) and immunostained with anti-GFAP ((a) and (b)), anti-S100B ((c) and (d)), and anti-Cx43 ((e) and (f)) antibodies (as indicated). The RCCS G-aggregate sections were also stained with propidium iodide (PI). Insets in (b), (d), and (f) show image magnification. Scale bars, 25 *μ*m.

To analyse the expression of the GL15 cell specific phenotype in the G-aggregates cultured in the RCCS bioreactor for up to 2 weeks, immunostaining for glial markers was carried out. The G-aggregates showed glial-cell-specific protein expression similar to that observed in the GL15 cells cultured as monolayers under 2D conditions (Figure 3). The G-aggregates and the GL15 cells cultured as monolayers both showed cytoplasmic localisation of GFAP and S100B, as two markers of the glial cytoskeleton (Figures 3(a)–3(d)).

Cell interactions due to gap-junction-mediated intercellular communication have been shown to have crucial roles in the regulation of the glial-cell network and nervous system functions [25]. For this reason, the expression of Cx43 was also investigated, as Cx43 is the main gap-junction protein expressed by astrocytes. As shown in Figure 3(e), the GL15 cells grown in two dimensions expressed Cx43 near the plasmalemma, at cell-cell contact areas, and in the cytoplasm. A similar distribution was also seen for Cx43 in the G-aggregates (Figure 3(f)).

#### 3.2.2. SH-SY5Y Cells

**Figure 4:**
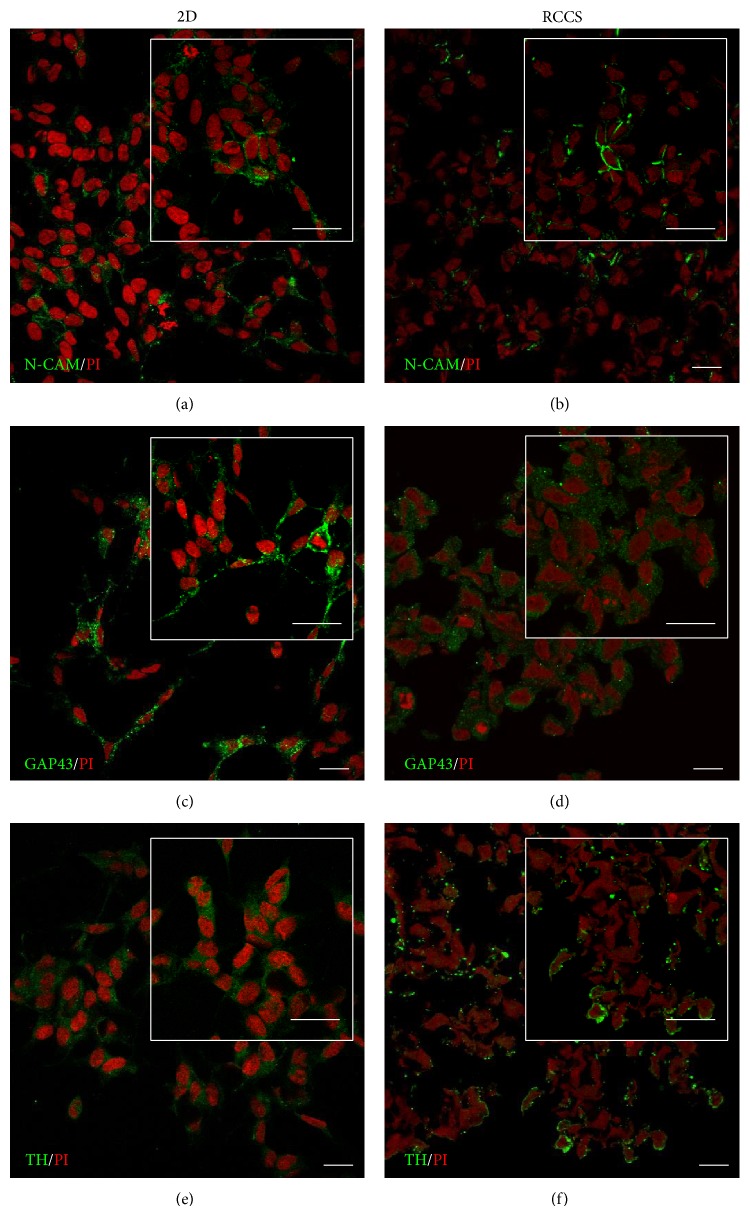
Neuronal marker localisation in SH-SY5Y cells. Representative confocal images of SH-SY5Y cells cultured as a monolayer (2D, (a), (c), and (e)) and under the modelled microgravity (RCCS bioreactor, (b), (d), and (f)) and immunostained with anti-N-CAM ((a) and (b)), anti-GAP43 ((c) and (d)), and anti-tyrosine hydroxylase (TH) ((e) and (f)) antibodies (as indicated). All of the cells were also stained with propidium iodide (PI). Insets show image magnification. Scale bars, 20 *μ*m.

We characterised the phenotype expressed by the SH-SY5Y cells in the S-aggregates maintained in the dynamic 3D culture in the RCCS bioreactor for 2 weeks, by determining the expression of the neuronal specific markers N-CAM, GAP43, and tyrosine hydroxylase. Immunofluorescence analysis revealed that N-CAM in the S-aggregates was localised towards the plasma membrane and near cell-cell contact areas, thus resembling its distribution in the SH-SY5Y cells cultured in 2D monolayers (Figures 4(a) and 4(b)), which showed cell-cell adhesion interactions. GAP43 is involved in neurite outgrowth and neuronal plasticity [26], and in SH-SY5Y cell monolayers it was localised into neurite-like processes (Figure 4(c)). In the S-aggregates, GAP43 was localised in the cytoplasmic compartment (Figure 4(d)). The distribution of tyrosine hydroxylase (TH), which is a rate-limiting enzyme in dopamine/norepinephrine synthesis [27], was in the cytoplasm under both of these cell-culture conditions (Figures 4(e) and 4(f)).

### 3.3. Quantitative Analysis of Phenotype-Specific Markers

#### 3.3.1. GL15 Cells

**Figure 5:**
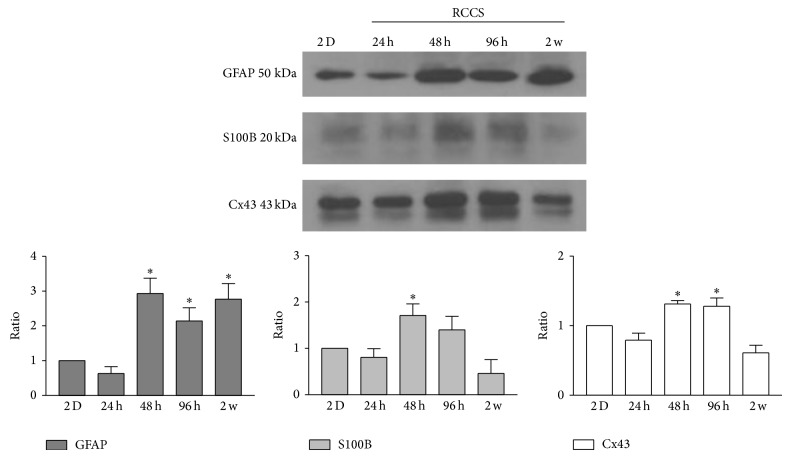
Expression of glial cell markers. Representative Western blotting and quantification of the levels of GFAP, S100B, and Cx43 in GL15 cells cultured in 2D monolayers and in the RCCS bioreactor for 24 h, 48 h, 96 h, and 2 weeks (2 w). Data are from densitometric ratio analyses as means ± SEM from 3 independent experiments. ^*^ *P* < 0.05 versus 2D monolayers.

The differentiation status of cells is characterised not only by marker localisation but also by marker expression levels. To evaluate potential quantitative differences between the G-aggregate modelled microgravity-exposed cultures and the GL15 cells as 2D static monolayer cultures, the expression levels of the GFAP, S100B, and Cx43 proteins were determined by Western blotting (Figure 5). The G-aggregates showed increased levels of GFAP, S100B, and Cx43 after the first 48 h of culture. These levels gradually decreased over the following 2 weeks, when those of S100B and Cx43 were similar to those observed in the GL15 cells as 2D static cultures, while those of GFAP remained increased in the G-aggregates (Figure 5).

#### 3.3.2. SH-SY5Y Cells

**Figure 6:**
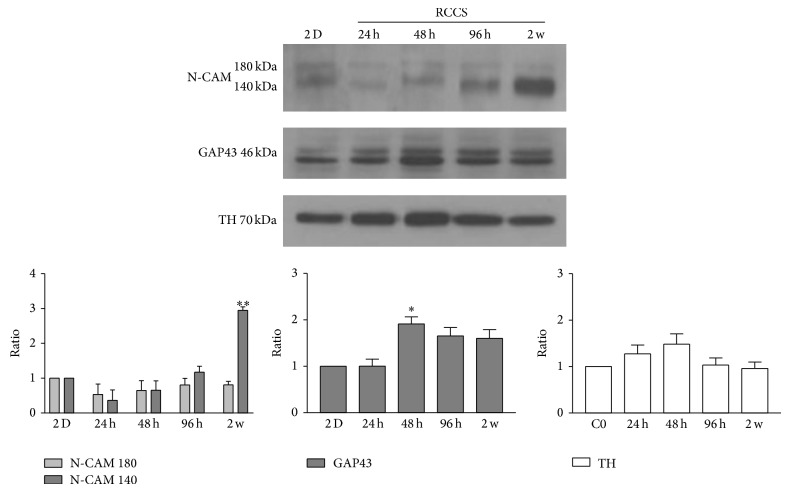
Expression of neuronal cell markers. Representative Western blotting and quantification of the levels of N-CAM, GAP43, and tyrosine hydroxylase (TH) in SH-SY5Y cells cultured in 2D monolayers and in the RCCS bioreactor for 24 h, 48 h, 96 h, and 2 weeks (2 w). Data are densitometric ratio analyses as means ±SEM from 3 independent experiments. ^*^ *P* < 0.05 and ^**^ *P* < 0.01 versus 2D monolayers.

Western blotting carried out for the S-aggregates showed that N-CAM-140 and GAP43 expression levels were increased during the incubation, compared to the SH-SY5Y cells as 2D static monolayer cultures (Figure 6). In particular, for the S-aggregates, N-CAM-140 reached a peak after 2 weeks, while GAP43 peaked after 48 h. Conversely, N-CAM-180 and tyrosine hydroxylase did not significantly change in the S-aggregates compared to SH-SY5Y cells as 2D static cultures (Figure 6).

### 3.4. Coculture of GL15 and SH-SY5Y Cells in the RCCS Bioreactor

**Figure 7:**
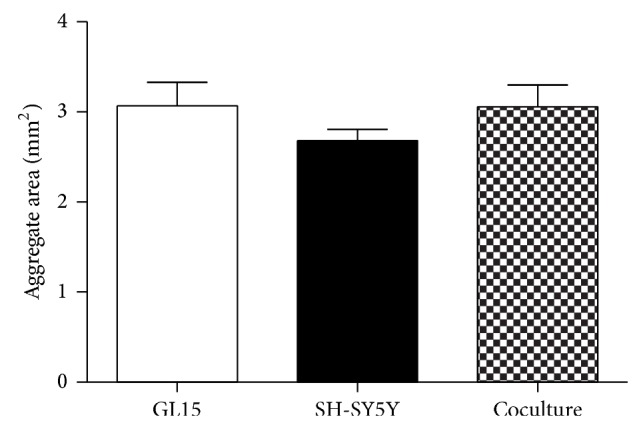
Cell aggregate sizes. Quantification of section area of GL15, SH-SY5Y, and cocultured (GL15 plus SH-SY5Y) cell aggregates (as indicated). Data are means ±SEM (*n* = 15) for the averaged areas of the aggregate sections, calculated using the ImageJ software (http://imagej.nih.gov/ij/).

The SH-SY5Y cells were also cocultured with the GL15 cells in the RCCS bioreactor with the aim to reestablish a more neural-like microenvironment and thus to be closer to* in vivo* conditions. Initial experiments were carried out to determine if it was possible to establish viable GL15 plus SH-SY5Y cocultures in the RCCS bioreactor, henceforth referred to as GS-aggregates. GL15 and SH-SY5Y cells were thus cocultured in the RCCS bioreactor at a 1 : 1 ratio for up to 2 weeks. At the end of this period, the sizes of the GS-aggregates were similar to those of the monotypic G-aggregates and S-aggregates (Figure 7), and although the S-aggregates appeared smaller than the others, these differences did not reach significance. Cell viability assays also showed that the GS-aggregates had low levels of apoptotic and necrotic cells (data not shown).

**Figure 8:**
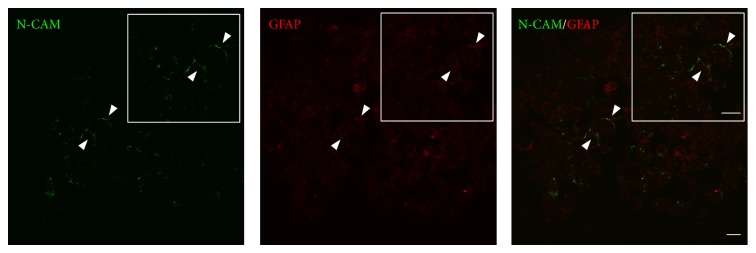
Localisation of glial and neuronal cell markers in the GS-aggregates. Representative confocal images of GS-aggregates cultured under the modelled microgravity, for 2 weeks, and immunostained with anti-N-CAM and anti-GFAP antibodies (as indicated). Insets show image magnification. Scale bars, 20 *μ*m.

To characterise the cell phenotype in these GS-aggregates, immunostaining was carried out for N-CAM, GFAP, and Cx43. These coculture conditions induced the establishment of GS-aggregates that contained both glial-like and neuronal-like cell phenotypes. After the 2 weeks of culture in the RCCS bioreactor, these GS-aggregates showed specific fluorescence signals for astrocyte (GFAP-positive) and neuronal (N-CAM-positive) phenotypes (Figure 8).

**Figure 9:**
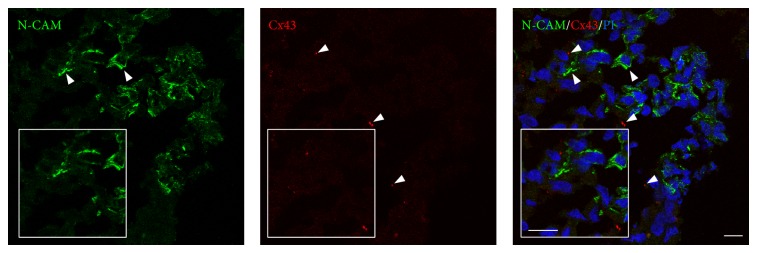
Localisation of cell-cell interaction markers in the GS-aggregates. Representative confocal images of GS-aggregates cultured under modelled microgravity, for 2 weeks, and immunostained with anti-N-CAM and anti-Cx43 antibodies (as indicated). The GS-aggregate sections were also stained with propidium iodide (PI; right). Insets show image magnification. Scale bars, 10 *μ*m.

The GS-aggregates were double-stained for N-CAM and Cx43. These N-CAM-specific and Cx43-specific fluorescent signals revealed a particular distribution of these proteins, whereby even if colocalisation of the N-CAM and Cx43 patterns was not evident, possible heterotypic cell-cell interactions could not be excluded. In particular, within the GS-aggregates, N-CAM localised to the peripheral areas of the cells, while Cx43-specific fluorescent spots appeared to be sparsely distributed, which indicated a low level of cell-cell functional interactions (Figure 9). In addition, in the same GS-aggregates, there were also evident N-CAM-negative and/or Cx43-negative cells, which indicated potential different cell activities due to different protein expression levels.

**Figure 10:**
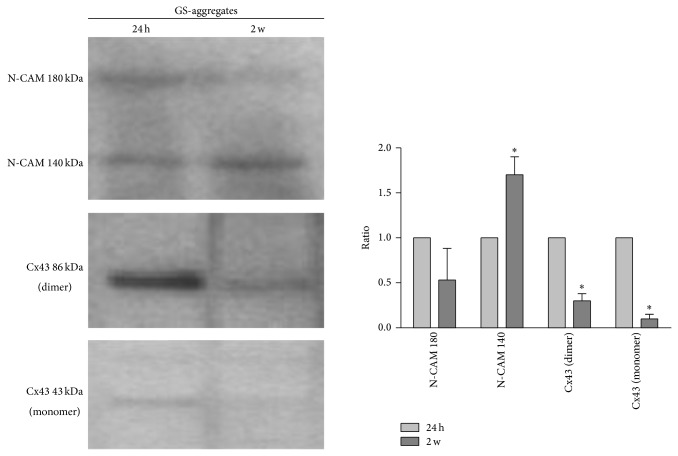
Expression levels of functional markers in the GS-aggregates. Representative Western blotting and quantification of the levels of N-CAM and Cx43 in GS-aggregates cultured in the RCCS bioreactor for 24 h and 2 weeks (2 w). Data are densitometric ratio analyses as means ±SEM from 3 independent experiments. ^*^ *P* < 0.05, for 2-week versus respective 24 h cultures.

Western blotting of N-CAM and Cx43 expression levels revealed that, in the GS-aggregates, the monomeric Cx43 protein (43 kDa) was downregulated during the RCCS bioreactor incubation. Interestingly, homogenates from the GS-aggregates showed a Cx43-positive band at 86 kDa, which demonstrates the presence of a dimeric form of Cx43, which was highly expressed in the initial phases of the coculture (over the first 24 h), and which significantly decreased over the 2 weeks of the GS-aggregates in the RCCS bioreactor (Figure 10). The N-CAM isoform expression pattern showed a slight, but not significant, decrease in N-CAM-180 levels and a significant increase in N-CAM-140 levels (Figure 10), which resembled the N-CAM-140 increase observed in the S-aggregate homogenates.

## 4. Discussion

There are evidences available showing that microgravity can affect the functioning of the nervous system, although the possible physiological mechanisms of these effects remain difficult to determine [7, 28]. Such difficulties in investigations into microgravity effects are mainly due to the poor models that are available, either because of their high cost and low availability (e.g., spaceflight) or because they are little representative of true microgravity conditions (e.g., hindlimb suspension/disuse model). Among the ground-based models,* in vitro* culture of cells/tissues within clinorotation-based systems (e.g., random positioning machine, RCCS bioreactor) represents a reasonable alternative to spaceflight. The RCCS bioreactor, in particular, was initially developed by NASA engineers to maintain cells in culture during space missions and to counteract the forces faced during shuttle launch and landing. The RCCS bioreactor was further used to maintain cells in dynamic 3D culture on the ground, and because of its particular properties, the RCCS bioreactor also allows the modelling of microgravity on the ground. Setting standardised parameters, it is possible to also promote the colocalisation of cells, the establishment of cell-cell contacts, and, consequently, the spontaneous formation of multicellular aggregates [11, 13]. Moreover, the rotation speed can also be regulated in such a way that it is possible to reach a vector-averaged gravity that simulates low-gravity conditions [14].

In the present study, we designed and investigated a powerful human-derived 3D organotypic-like model of nervous system tissue. The experimental strategy was to study this 3D cell aggregation in terms of the cell phenotypes following short-term culture (up to 48 h, as a time that allows the formation of multicellular aggregates) and long-term (up to 2 weeks) culture, to analyse the effects of this modelled microgravity system on cell behaviour. However, apart from the effects related to microgravity, the development of a reliable neuro/glia cell* in vitro* model is of great interest for basic and clinical research in the field of the nervous system. Thus, we developed astrocyte-like and neuron-like* in vitro* models here, as 3D monotypic (GL15 cells only; SH-SY5Y cells only) and heterotypic (cocultures of both GL15 and SH-SY5Y cells) cell cultures in the RCCS bioreactor.

The particular dynamic conditions in the RCCS bioreactor have been shown to favour the differentiated phenotype expression for numerous cell and tissue types [13, 24, 29–31]. In our hands, over 48 h of culture, these optimal dynamic conditions favoured spontaneous formation of healthy multicellular aggregates according to the cell type considered, as demonstrated by the low cell death in these spontaneous cell aggregates. The survival of these G-aggregates and S-aggregates and also of the GS-aggregates was assessed for up to 2 weeks in the RCCS bioreactor cultures, and the data confirm the absence of significant necrosis in their central cores, in contrast to what has been reported in the literature for similar static culture conditions [32]. This evidence supported our choice to use the spontaneously formed aggregate method, as this allowed the random distribution of the cells inside the aggregates, which is a feature that is particularly important for the establishment of the heterotypic coculture model.

Under our 3D cell culture conditions in growth medium, the GL15 cells showed an astrocyte-like phenotype, with the expression of the glial-specific markers GFAP [33] and S100B. Interestingly, under these conditions, Cx43 expression was also evident in these G-aggregates. These data confirm the importance of cell-cell interactions in the regulation of phenotypic expression. The modulation of Cx43 expression might be related to the formation of these G-aggregates in the 3D culture. During the first phase of G-aggregate formation, there was upregulation of Cx43 expression. In a previous study, we showed that these GL15 cells express Cx43 and form junctional channels where the permeability is directly related to the cell proliferation rate, as it decreased when their differentiated status was reached [16]. In the present study, this transient upregulation of Cx43 during G-aggregate formation might support the hypothesis that Cx43 has a crucial role and function in cellular aggregation in addition to its well-known involvement in differentiation processes [34]. This hypothesis was also supported by Cotrina and colleagues, who demonstrated a role for Cx43 hemichannels in cellular adhesion of C6 glioma cells [35].

The optimal dynamic culture conditions provided by the RCCS bioreactor were also demonstrated by the favoured expression of neuronal-specific markers by the SH-SY5Y cells in the S-aggregates, such as tyrosine hydroxylase, GAP43, and N-CAM. The expression levels of tyrosine hydroxylase appeared similar in both the 2D and the 3D cultures at all of the times tested, which demonstrated the adrenergic phenotype that was expressed by these S-aggregates. GAP43 expression increased during the cell aggregation (48 h), which confirms active cell-cell interactions, with the cytoskeletal modifications shown by GAP43 regulation. The stabilisation of these cell aggregates is supported by the increase in N-CAM expression.

During the long-term exposure to modelled microgravity, specific protein expression was differently regulated in the cell aggregates. Even after 2 weeks under culture in the modelled microgravity, in the G-aggregates the glial-specific and functional markers (i.e., GFAP, S100B, and Cx43) showed localisation patterns that were similar to those observed in the monolayers under normal gravity conditions. Interestingly, under microgravity, S100B and Cx43 expression levels in the G-aggregates were downregulated over two weeks, as compared to those in the G-aggregate cultures at 48 h exposure, whereas there was a slight transient, although not significant, effect on GFAP expression.

The exact physiological roles of GFAP in astrocytes remain incompletely understood, although they appear to be involved in cell-shape maintenance, nervous system cytoarchitecture, mechanical stability, and synaptic function [36]. On the other hand, it is well-known that Cx43 modulation is involved in neuronal development and plasticity [37] and that S100B is expressed and also secreted by astrocytes and can thus be an extracellular mediator of cell signalling [38]. This evidence supports the hypotheses that the microgravity can affect not only cell shape but also cell function.

In the S-aggregates, the modelled microgravity conditions did not have any significant effects on the localisation of N-CAM and tyrosine hydroxylase, but they were shown to induce a switch of the GAP43 protein from the neurite-like processes to the cytoplasmic compartment. In addition, the microgravity induced a slight, although not significant, decrease in the expression levels of GAP43 and tyrosine hydroxylase, while it had no effect on the expression of the N-CAM-180 isoform but significantly increased the expression of the N-CAM-140 isoform, which has been shown to have a key role in neuronal survival and signal transduction [39, 40]. This suggests the involvement of N-CAM-140 during this modelled microgravity exposure that could promote a significant degree of neuronal remodelling and survival.

It has been previously reported that, in neuro/glial cocultures, the neurons induce a reduction in astrocyte proliferation [41]. In particular, this effect was mediated by membrane-membrane interactions between the neurons and the astroglia* in vitro* and raised the possibility that membrane elements involved in glial cell growth regulation include neuron-glial interaction molecules [41]. In our neuron-like and glial-cell-like coculture, we focused our attention on N-CAM and Cx43 expression, as these participate in important intercellular signal interactions. The GL15 cells used here did not express N-CAM isoforms (data not shown), and the immunofluorescence signals might reveal SH-SY5Y homotypic interactions, even if N-CAM heterotypic interactions could not be excluded (such as N-CAM-integrins). However the N-CAM expression pattern in homogenates from GS-aggregates resembled that for the S-aggregates, with an increase in the expression of N-CAM-140, one of the three main isoforms of N-CAM that is implicated in regeneration and remodelling of the nervous system [42].

Gap-junction-mediated intercellular communication among astrocytes has long been thought to contribute to tissue homeostasis in the brain [43]. Cx43 has been used as a marker to investigate neuron-glia interactions [22]. Astrocytes express Cx30 and Cx43, which can form homotypic (Cx43/Cx43 and Cx30/Cx30), but not heterotypic, junctions [44]. Interestingly, in the homogenates from the GS-aggregates, in addition to the classical 43 kDa form of Cx43, a dimeric form of Cx43 (i.e., 86 kDa) was also expressed. This Cx43 dimeric form has been related to stress conditions. In other models, oxidative stress status has been related to the appearance of such a higher molecular weight band for Cx43, which suggests that this represents an aggregated form of Cx43 [45]. Under our conditions, the presence of this dimeric form of Cx43 might reveal a first phase of impact between the neuronal-like and the glial-like phenotypes; subsequently, these Cx43 forms significantly decreased when the GS-aggregates reached dynamic adaptive conditions.

## 5. Conclusions

In conclusion, the evidence presented here suggests that the 3D laminar flow, the high mass transfer, and the low shear-stress microenvironment generated by the RCCS bioreactor represent optimal conditions for the well-being of monotypic neural-like and glial-like cells, as well as for heterotypic aggregates, and for long-term culture. Moreover, such model system can reproduce 3D cell-cell interactions that are similar to those under* in vivo* conditions [46] and can mimic the microgravity conditions of exposure. Our data highlight how some phenotypic markers of monotypic and heterotypic neuro/glia culture models can be influenced by microgravity.

The data presented here open a wide range of specific investigations in terms of cell transduction pathways, cell-cell interactions and signalling, and heterotypic culture biology, and the cell models we have described and analysed here represent important tools in the study of* in vitro* biological and pathological processes of the nervous system.
